# LncRNA HOTTIP impacts the proliferation and differentiation of fibroblast-like synoviocytes in ankylosing spondylitis through the microRNA-30b-3p/PGK1 axis

**DOI:** 10.1186/s13018-023-03653-4

**Published:** 2023-03-24

**Authors:** Li Wei, Xin Zhang, Yu Yao, Weizhuo Zheng, Jun Tian

**Affiliations:** Department of Orthopaedic Ward 1, The Second Affiliated Hospital of Harbin Medical University, 246 Xuefu Road, Harbin, 150000 Heilongjiang China

**Keywords:** Ankylosing spondylitis, Long non-coding RNA HOTTIP, MicroRNA-30b-3p, Phosphoglycerate kinase 1, Fibroblast-like synoviocytes, Proliferation, Differentiation

## Abstract

**Objective:**

Long non-coding RNAs (lncRNAs) and microRNAs (miRNAs) have been reported to exert regulatory effects on biological processes. This study intended to assess the role of the lncRNA HOXA transcript at the distal tip (HOTTIP)/miR-30b-3p/phosphoglycerate kinase 1 (PGK1) axis in ankylosing spondylitis (AS).

**Methods:**

Levels of HOTTIP, miR-30b-3p and PGK1 in AS synovial tissues and cultured AS fibroblast-like synoviocytes (ASFLSs) were assessed. The ASFLSs were identified and, respectively, treated with altered expression of HOTTIP and miR-30b-3p, and then, the proliferation and differentiation of the ASFLSs were assessed. The AS mouse models were established by injection of proteoglycan and Freund’s complete adjuvant and then were treated with altered expression of HOTTIP and miR-30b-3p, and the pathological changes and apoptosis of synoviocytes in mice’ synovial tissues were measured. The relationship of HOTTIP, miR-30b-3p and PGK1 was verified.

**Results:**

HOTTIP and PGK1 were elevated, while miR-30b-3p was reduced in AS synovial tissues and ASFLSs. Elevated miR-30b-3p or inhibited HOTTIP restrained proliferation and differentiation of ASFLSs and also improved the pathological changes and promoted apoptosis of synoviocytes in mice’s synovial tissues. PGK1 was a target of miR-30b-3p, and miR-30b-3p could directly bind to HOTTIP. Silencing miR-30b-3p or overexpressing PGK1 reversed the improvement of AS by knocking down HOTTIP or up-regulating miR-30b-3p.

**Conclusion:**

Our study suggests that reduced HOTTIP ameliorates AS progression by suppressing the proliferation and differentiation of ASFLSs through the interaction of miR-30b-3p and PGK1.

**Supplementary Information:**

The online version contains supplementary material available at 10.1186/s13018-023-03653-4.

## Introduction

Ankylosing spondylitis (AS) is a chronic inflammatory disease that mostly influences the sacroiliac joint, vertebrae and spinal ligaments [1, 2]. This disease often begins with enthesopathy in the axial skeleton below the atlantoaxial joint and progresses to ossification of spinal ligament, intervertebral disk, endplate and apophyseal structure. AS is characterized by inflammatory back pain, declined lumbar motility or reduced chest wall expansion [3]. Genetic studies and the impacts of local tissue factors, including biomechanical stress and bacterial products, are of great significance for a chronic innate immune response in AS [4, 5]. At present, no specific diagnostic technique is available for AS even though conventional diagnostic regimens such as laboratory tests, clinical symptoms and imaging techniques are utilized [6, 7]. The goal of AS management is to achieve remission, dominate disease activity and improve patient’s quality of life [8]. Hence, it is urgent to further seek novel targets for AS.

Long non-coding RNAs (lncRNAs) serve as both transcriptional activators and repressors consistent with diverse chromatin modifiers [9]. Some particular lncRNAs, such as NKILA [10] and lncRNA LINC00311 [11], have been implicated in AS development. The lncRNA HOXA transcript at the distal tip (HOTTIP) is transcribed from the 5’ tip of HOXA cluster [12], which has been demonstrated to be involved in osteosarcoma (OS) [13] and processes of endochondral ossification and osteoarthritic progression [14]. Nevertheless, no specific research has focused on the influence of HOTTIP in AS. MicroRNAs (miRNAs) are able to modulate protein expression through binding to the complementary sequence of target mRNA 3’-untranslation region (3’-UTR) [15]. As previously reported, miRNAs are involved in musculoskeletal conditions, such as tendon injuries and osteoarthritis [16, 17]. It has been demonstrated that several miRNAs are abnormally expressed in AS, such as miR-214 [18] and miR-451 [19]. The miR-30 family is comprised of 5 members, from miR-30a to miR-30e, and is evolutionarily conserved [20]. It has been revealed that miR-30b could regulate osteogenesis in bone marrow mesenchymal stem cells [21], and the miR-30 family has been found to be involved in osteoblastic and osteocytic differentiation from mesenchymal stem cells [22]. Additionally, miR-30b-5p has been detected to be up-regulated in radiographic axial spondyloarthropathy (rad-AxSpA) [23]. Nevertheless, the effect of miR-30b-3p on AS remains unexplored. Moreover, a recent study has demonstrated that HOTTIP could act as a molecular sponge to regulate miR-30b expression in esophageal squamous cell carcinoma (ESCC) [24], while their correlation in AS remains to be elucidated. Phosphoglycerate kinase 1 (PGK1) is a vital glycolytic enzyme that produces adenosine triphosphate through catalyzing the conversion of 1,3-diphosphoglycerate to 3-phosphoglycerate [25], which has been implicated in spinal muscular atrophy [26] and collagen-induced arthritis (CIA) [27]. However, the role of PGK1 in AS, as well as the relation between PGK1 and miR-30b-3p in human disease, has not been studied.

Our research concentrated on the role of the lncRNA HOTTIP/miR-30b-3p/PGK1 axis in the AS, and we supposed that HOTTIP could bind with miR-30b-3p to function in the proliferation and differentiation of fibroblast-like synoviocytes (FLSs) in AS by targeting PGK1.

## Materials and methods

### Ethics statement

The protocol of clinical experiments in this study was ratified by the Ethic Committee of The Second Affiliated Hospital of Harbin Medical University. Written informed consents were acquired from all participants. Animal experiments were approved by the Institutional Animal Care and Use Committee of The Second Affiliated Hospital of Harbin Medical University.

### Collection of clinical samples

Eighty-two cases of AS patients (54 males, 28 females, mean age of 56.8 years) that had accepted treatment in the Second Affiliated Hospital of Harbin Medical University were collected, and all the patients were in line with the New York criteria that revised by American College of Rheumatology in 1984 [28], and the samples were obtained by total hip replacement. A total of 82 patients with femoral neck fracture requiring open surgery or total hip replacement without AS were selected as the control group (50 males, 32 females, mean age of 58.6 years). The specimens were preserved in liquid nitrogen. No difference was observed in age and gender between patients in the AS group and the control group (*P* > 0.05). Clinical features and parameters are detailed in Additional file 1: Table S1.

### Culture of FLSs

Synovial tissues from AS patient were soaked in Hank’s solution and then cut into 1-mm^3^ synovial blocks and cultured for 24 h in fetal bovine serum (FBS)-contained medium. After being detached by 0.25% trypsin, the purified synoviocytes were continuously cultured at 37℃ with 5% CO_2_. The synoviocytes in the primary passage were not observed during the first 4 d and from the 5th d on, but the cell proliferation was observed everyday under a fluorescence microscope with the medium changed every 3 d. After the cells grew to cover the bottom of the flasks, they were detached by 0.25% trypsin, and the suspended synoviocytes were further cultivated until reaching the third passage for subsequent experiments. The normal human fibroblast-like synoviocytes (HFLSs) were obtained from American Type Culture Collection (VA, USA).

### Cell identification

The AS fibroblast-like synoviocytes (ASFLSs) in the third passage were seeded onto six-well plates with cover glass (24 mm × 24 mm, treated with polylysine/glycerin) at 1 × 10^5^ cells/well and cultured for 34 h at 37℃ with 5% CO_2_. After being fixed by 4% paraformaldehyde, the cells were permeabilized by 0.25% Triton X-100, sealed by 3% bovine serum albumin (BSA), cultivated with vimentin antibody at 4℃ overnight and rhodamine-labeled fluorescence secondary antibody and then photographed under a confocal microscope.

Phenotype measurement of ASFLSs: ASFLSs in the third passage were collected for cell surface molecular staining by using a flow cytometer.

### Cell grouping

The FLSs were classified into 10 groups: the blank group (HFLSs without any treatment), the ASFLS group (ASFLSs without any treatment), the small interfering RNA (si)-negative control (NC) group (ASFLSs were transfected with siRNA NC), the si-HOTTIP group (ASFLSs were transfected with HOTTIP siRNA), the mimic NC group (ASFLSs were transfected with mimic NC), the miR-30b-3p mimic group (ASFLSs were transfected with miR-30b-3p mimic), the si-HOTTIP + inhibitor NC group (ASFLSs were transfected with HOTTIP siRNA and inhibitor NC), the si-HOTTIP + miR-30b-3p inhibitor group (ASFLSs were transfected with HOTTIP siRNA and miR-30b-3p inhibitor), the miR-30b-3p mimic + oe-NC group (ASFLSs were transfected with miR-30b-3p mimic and empty pcDNA 3.1 vector) and the miR-30b-3p mimic + oe-PGK1 group (ASFLSs were transfected with miR-30b-3p mimic and pcDNA-PGK1 vector). The oligonucleotides, siRNA and plasmids were all purchased from GenePharma Co., Ltd. (Shanghai, China).

### Cell viability assay

The FLSs were seeded onto 96-well plates at 200 μL/well (10^4^–10^5^ cells/well), and the medium was changed to a fresh one when the cell confluence reached 60%. Next, each well was appended with 20 μL MTT solution (5 mg/mL) for 4-h incubation at 37℃. With the medium removed, each well was incubated with 150 μL dimethyl sulfoxide (DMSO) for 10 min without light exposure. The optical density (OD) value of each well at 570 nm was evaluated by a microplate reader.

### Alizarin red staining

The FLSs were seeded into 24-well plates with cover glass at 1 × 10^4^cells/well (total volume was 500 μL), the medium was changed into 1 mL mineralization induction medium containing 0.1 μl/L dexamethasone + 10 mmol/L β-glycerophosphate + 50 μl/L ascorbic acid after adherence and the cover glass was taken out when there appeared round or oval nodules, which were then fixed for 30 min with 75% ethanol, dyed by alizarin red dye for 3–5 min, dehydrated by gradient ethanol, permeabilized by xylene and blocked by neutral balsam. After being photographed, the extracellular calcified nodules were analyzed by ImageJ software.

### Experimental animals

BALB/c mice (aging 6 w) were fed in specific pathogen-free rooms with free access to water and food.

The AS mouse models were established by intraperitoneally injected with proteoglycan and Freund’s complete adjuvant (FCA) (75 μg proteoglycan and 150 μL FCA). One week after the injection, the mice were conducted with immunity strengthening (FCA was replaced by Freund’s incomplete adjuvant in the first immunization) [29].

### Animal grouping

Mice were randomly classified into 10 groups (n = 10): the normal group (mice without treatment), the model group (the modeled mice were injected with 1 × PBS), the si-NC group (the modeled mice were injected with 5 μL adenovirus silencing HOTTIP NC (5 × 10^9^ viral particles/μL)) [30], the si-HOTTIP group (the modeled mice were injected with adenovirus silencing HOTTIP (5 × 10^9^ viral particles/μL)), the agomir NC group (the modeled mice were injected with adenovirus overexpressing miR-30b-3p NC (5 × 10^9^ viral particles/μL)), the miR-30b-3p agomir group (the modeled mice were injected with adenovirus overexpressing miR-30b-3p (5 × 10^9^ viral particles/μL)), the si-HOTTIP + antagomir NC group (the modeled mice were injected with adenovirus silencing HOTTIP and adenovirus inhibiting miR-30b-3p NC (both 5 × 10^9^ viral particles/μL)), the si-HOTTIP + miR-30b-3p antagomir group (the modeled mice were injected with adenovirus silencing HOTTIP and adenovirus inhibiting miR-30b-3p (both 5 × 10^9^ viral particles/μL)), the miR-30b-3p agomir + oe-NC group (the modeled mice were injected with adenovirus overexpressing miR-30b-3p and adenovirus overexpressing PGK1 NC (both 5 × 10^9^ viral particles/μL)) and the miR-30b-3p agomir + oe-PGK1 group (the modeled mice were injected with adenovirus overexpressing miR-30b-3p and adenovirus overexpressing PGK1 (both 5 × 10^9^ viral particles/μL)). All of the adenoviruses were obtained from RiboBio Co., Ltd. (Guangdong, China), and were performed intra-articular injection into mice, once weekly after the first immunization for a total of 6 weeks.

### Sampling

On the 42nd d of the first immunization, blood was collected from mice’ orbit and centrifuged at 4℃, and the serum was preserved at −20℃. After being euthanized, the sacroiliac joints were extracted from the mice, which were sectioned, fixed in 4% paraformaldehyde for 12 h, regularly decalcified, dehydrated, permeabilized, embedded and performed with hematoxylin–eosin (HE) staining and terminal deoxynucleotidyl transferase-mediated deoxyuridine triphosphate (dUTP) nick end-labeling (TUNEL) staining, and the others were kept at −80℃ for RT-qPCR.

### Enzyme-linked immunosorbent assay (ELISA)

The contents of serum inflammatory factors TNF-α, interleukin (IL)-6 and IL-β were determined by the ELISA kits (R&D Systems, MN, USA). The contents of these factors were calculated according to the OD value of the samples for the plotting of the standard curve.

### HE staining

The paraffin sections were heated at 60℃, dehydrated by ethanol, permeabilized by xylene, dyed by hematoxylin, differentiated by 1% hydrochloric alcohol and reacted with 1% ammonium hydroxide. After that, the sections were counterstained by 1% eosin solution for 5 min, routinely dehydrated, permeabilized, dried and blocked. A light microscope was implemented to observe the pathological structure of the articulationes sacroiliaca.

### TUNEL staining

The TUNEL staining was following the directions of in situ cell apoptosis detection kits (Roche, Shanghai, China). In detail, the paraffin sections were dewaxed with xylene and then hydrated by ethanol and supplemented with proteinase K (20 g/mL). Subsequently, the sections were supplemented with TUNEL reaction mixture, followed by staining of the nuclei with DAPI. Five random fields were selected using an IX71 fluorescence microscope (Olympus, Tokyo, Japan) and calculated for apoptosis rate.

### RT-qPCR

Total RNA was extracted using TRIzol reagent (Invitrogen, Carlsbad, CA, USA). Following the instructions of All-in-One™ miRNA First-Strand cDNA Synthesis Kit (Boyi Biotechnology Co., Ltd., Shanghai, China) and PrimeScript™ RT Reagent Kit (Takara Bio, Inc.), the RNA was reversely transcripted into cDNA, which was then conducted with qPCR by using SYBR Green PCR Kit. U6 and GAPDA were taken as the loading controls. The data were analyzed by the 2-^△△Ct^ method [31]. The RNA primers for genes (Additional file 1: Table S2) were designed and synthetized by Invitrogen (Shanghai, China).

### Western blot analysis

The total proteins were extracted by radio-immunoprecipitation assay reagent (Gibco, Grand Island, NY, USA), the concentrations of which were evaluated by BCA kits (Bio-Rad Laboratories, CA, USA). The proteins were conducted with sodium dodecyl sulfate–polyacrylamide gel electrophoresis for 2 h and subsequently transferred onto membranes, followed by 5-min blocking with 5% skim milk powder. Subsequently, the membranes were supplemented with primary antibodies PGK1 (1: 1000) and GAPDH (1: 1000) (both from Santa Cruz Biotechnology Inc., CA, USA) for incubation at 4℃ overnight, followed by 1-h incubation with the corresponding secondary antibody. The gray value was analyzed by Image Lab software.

### Dual-luciferase reporter gene assay

HOTTIP or PGK1 3’UTR segment covering wild-type (Wt) and mutant-type (Mut) miR-30b-3p-binding site was inserted into pmirGLO dual-luciferase reporter vector (Promega, MI, USA) to obtain pmirGLO-HOTTIP-Wt/Mut and pmirGLO-PGK1-Wt/Mut. The WT (or Mut) pmirGLO luciferase reporter gene vector of HOTTIP (or PGK1 3’UTR) and miR-30b-3p mimic (or mimic NC) were co-transfected into cells with Lipofectamine 3000 (Invitrogen). The luciferase activity was analyzed using a dual-luciferase reporter gene detection system (Promega).

### RNA pull-down assay

Biotin-labeled WT miR-30b-3p (Bio-miR-30b-3p-WT), MUT miR-30b-3p (Bio-miR-30b-3p-MUT) and Bio-NC were designed and synthetized. The extraction of the total RNA was realized by TRIzol, and then, HOTTIP expression was evaluated by RT-qPCR [32].

### Statistical analysis

SPSS 21.0 software (IBM Corp., Armonk, NY, USA) was utilized for data analysis. Data were depicted as mean ± standard deviation. The t-test and one-way analysis of variance (ANOVA) followed by Tukey’s post hoc test were implemented for group comparisons. *P* value less than 0.05 was indicative of statistically significant difference.

## Results

### Identification of ASFLSs

**Fig. 1:**
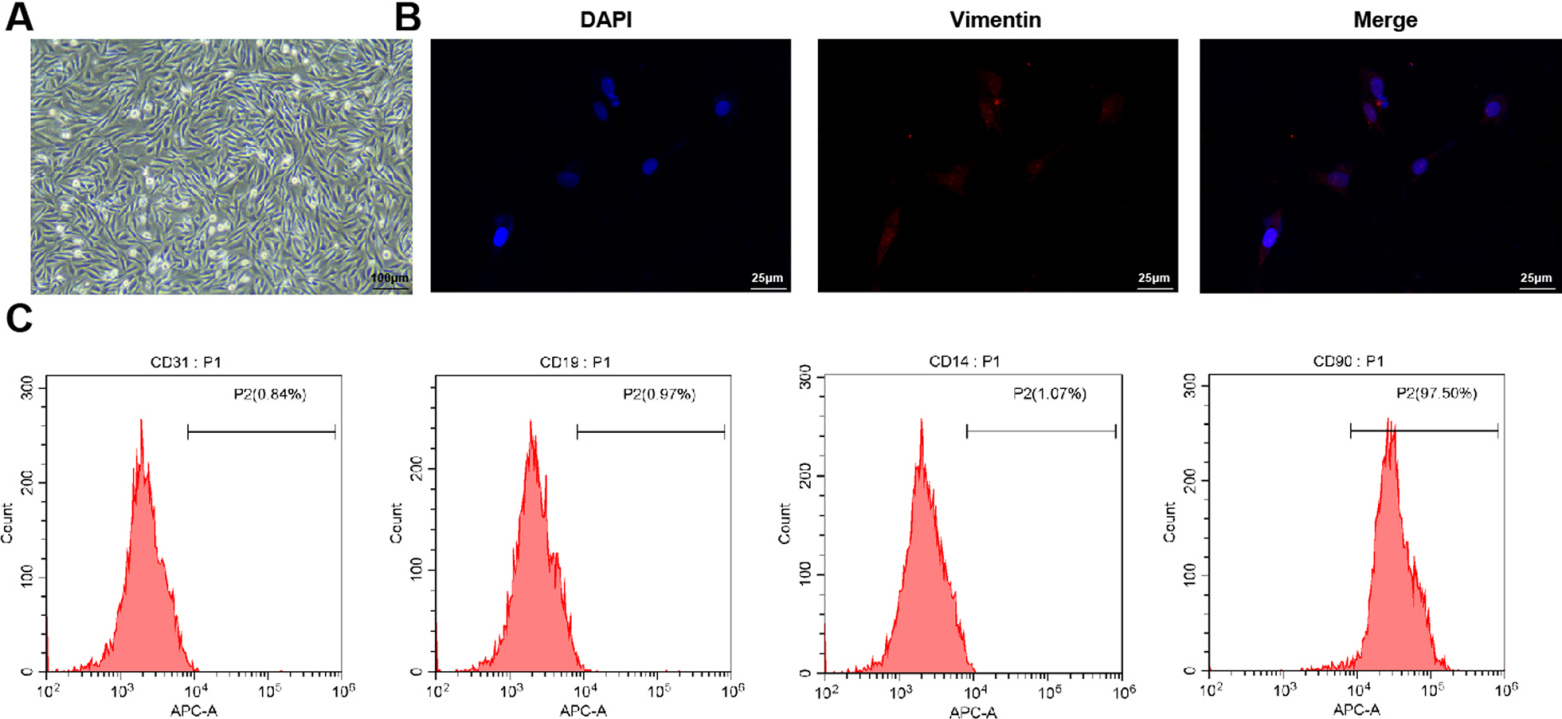
Identification of ASFLSs. **A** Primary ASFLSs were observed under an inverted microscope. **B** Immunofluorescence images of anti-vimentin. **C** Expression of CD31, CD19, CD14 and CD90 on the surface of ASFLSs was assessed by flow cytometry

For elucidating the occurrence and development of synovial FLSs in AS patients, the cells were isolated from synovial tissues by tissue culture method and conducted with primary culture. An inverted microscope was implemented to observe whether the cells were successfully isolated and cultured and it was revealed that the cells in or after the third passage were fusiform (Fig. 1A). The vimentin antibodies in cytoplasm represented red fluorescence, and there were a small number of fluorescence in nuclei, which was positive, and the nuclei DNA that combined with DAPI showed as blue fluorescence (Fig. 1B). As analyzed by a flow cytometer, the positive rate of CD90 expression on the surface of ASFLSs was 97.5 ± 1.5%, and that of CD14, CD19 and CD31 severally was 1.07 ± 0.5%, 0.97 ± 0.1% and 0.84 ± 0.1%, respectively (Fig. 1C). These outcomes suggested that the cultured cells were ASFLSs.

### HOTTIP and PGK1 are up-regulated in AS, and down-regulated HOTTIP impedes the proliferation and differentiation of ASFLSs

**Fig. 2:**
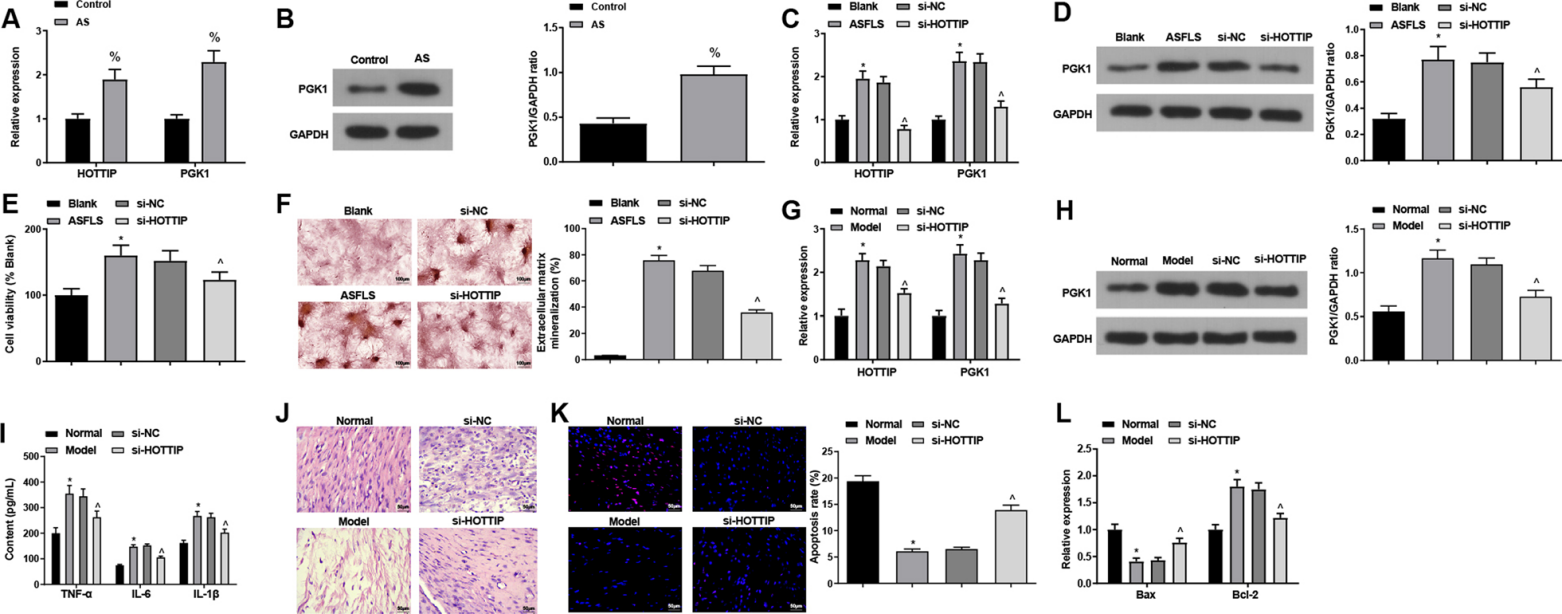
HOTTIP and PGK1 are up-regulated in AS, and down-regulated HOTTIP impedes the proliferation and differentiation of ASFLSs. **A**, **B** Expression levels of HOTTIP and PGK1 in AS patients. **C**, **D** Expression levels of HOTTIP and PGK1 in ASFLSs. **E** Proliferation rate of ASFLSs was measured through MTT assay. **F** The extracellular calcified nodules of ASFLSs were tested by Alizarin red staining. **G**, **H** Expression levels of HOTTIP and PGK1 in AS mice. **I** Contents of serum TNF-α, IL-6 and IL-β in each group. **J** Representative images of HE staining. **K** Representative images of TUNEL staining and the apoptosis rate of synoviocytes in each group. **L** Expression levels of Bax and Bcl-2 in synovial tissues. % *P* < 0.05 *vs* the control group, *P < 0.05 *vs* the blank or normal group, ^ *P* < 0.05 *vs* the si-NC group

The evaluation of the expression levels of HOTTIP and PGK1 in AS patients disclosed that the expression levels of HOTTIP and PGK1 in the synovial tissues were increased (Fig. 2A, B).

The next step was to examine the transfection efficiency and effect of HOTTIP and PGK1 on ASFLSs, with the results demonstrating that the expression levels of HOTTIP and PGK1 in ASFLSs were elevated in comparison with HFLSs, while the levels of them were reduced in ASFLSs treated with si-HOTTIP, indicating that the transfection was successful (Fig. 2C, D).

The subsequent MTT assay and Alizarin red staining revealed that enhanced cell proliferation and elevated percentage of extracellular calcified nodules were observed in ASFLSs, without significant extracellular calcified nodules formed. In ASFLSs treated with si-HOTTIP, there were diminished cell proliferation and reduced percentage of extracellular calcified nodules (Fig. 2F).

In addition, in order to further disclose the effect of knockdown of HOTTIP on AS, we established an AS mouse model, which validated that the model establishment was successful (Fig. 2G, H). The contents of serum inflammatory factors in mice revealed that the levels of TNF-α, IL-6 and IL-1β in the serum of AS mice were enhanced, which were reduced after inhibiting HOTTIP (Fig. 2I). The observations of HE staining presented that the joints of the mice in the normal group showed normal morphology and structure of tissues without inflammatory cell infiltration; in the model group and si-NC group, the inflammation and hyperplasia of the sacroiliac joints in mice were evident compared with the normal group, mainly manifested by a large number of inflammatory cell and synovial cell infiltration, and the synovial tissue was significantly thickened; inflammatory cells were also seen in the si-HOTTIP group, but it was improved versus the model group (Fig. 2J). The results of TUNEL staining and PCR assay suggested that the apoptosis rate of synovial cells was reduced, accompanied by reduced Bax and elevated Bcl-2 in rats of the model group; inhibition of HOTTIP resulted in promoted apoptosis of synoviocytes, accompanied by elevated Bax and reduced Bcl-2 (Fig. 2K, L).

The above results imply that silencing HOTTIP represses the proliferation and differentiation of ASFLSs in AS, thereby reducing the proliferation of synovial tissues and improving the symptoms of AS.

### miR-30b-3p is down-regulated in AS, and up-regulated miR-30b-3p impedes the proliferation and differentiation of ASFLSs

**Fig. 3:**
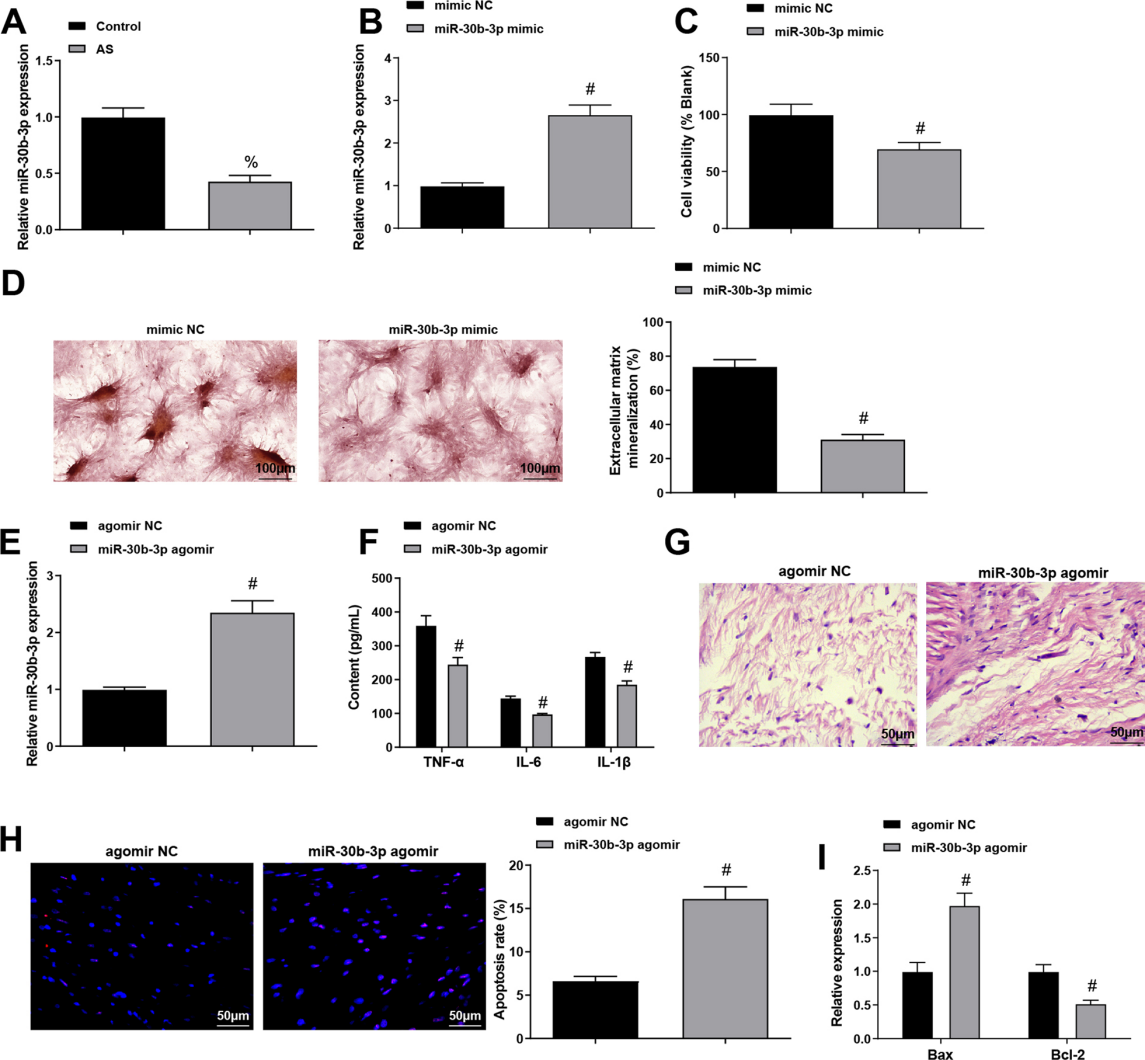
miR-30b-3p is down-regulated in AS, and down-regulated miR-30b-3p impedes the proliferation and differentiation of ASFLSs. **A** Expression level of miR-30b-3p in AS patients. **B** Expression level of miR-30b-3p in ASFLSs. **C** Proliferation rate of ASFLSs was measured through MTT assay. **D** The extracellular calcified nodules of ASFLSs were tested by Alizarin red staining. **E** Expression level of miR-30b-3p in AS mice. **F** Contents of serum TNF-α, IL-6 and IL-β in each group. **G** Representative images of HE staining. **H** Representative images of TUNEL staining and the apoptosis rate of synoviocytes in each group. **I** Expression levels of Bax and Bcl-2 in synovial tissues. % *P* < 0.05 *vs* the control group, # *P* < 0.05 vs. the mimic NC or agomir NC group

Next, miR-30b-3p level in patients with AS was determined, which revealed a reduced miR-30b-3p level in AS patients (Fig. 3A).

For further investigating the effect of miR-30b-3p on ASFLSs, we transfected cells with miR-30b-3p mimic and its NC, and PCR assay indicated that the transfection was successful (Fig. 3B). The subsequent MTT assay and Alizarin red staining revealed that in ASFLSs treated with miR-30b-3p mimic, there were diminished cell proliferation and reduced percentage of extracellular calcified nodules (Fig. 3C, D).

In addition, with the aim to further elucidate the impact of miR-30b-3p on AS, we established an AS mouse model, which validated that the model establishment was successful (Fig. 3E). The contents of serum inflammatory factors in mice revealed that the levels of TNF-α, IL-6 and IL-1β in the serum of AS mice were reduced after up-regulating miR-30b-3p (Fig. 3F). The observations of HE staining presented that significant improvement in inflammatory infiltration was observed in mice treated with miR-30b-3p agomir (Fig. 3G). The results of TUNEL staining and PCR assay suggested that miR-30b-3p agomir resulted in promoted apoptosis of synoviocytes, accompanied by elevated Bax and reduced Bcl-2 (Fig. 3H, I).

It is concluded that up-regulating miR-30b-3p impedes the proliferation and differentiation of ASFLSs in AS, thereby reducing the proliferation of synovial tissues and improving the symptoms of AS.

### HOTTIP negatively regulates miR-30b-3p and PGK1 is targeted by miR-30b-3p

**Fig. 4:**
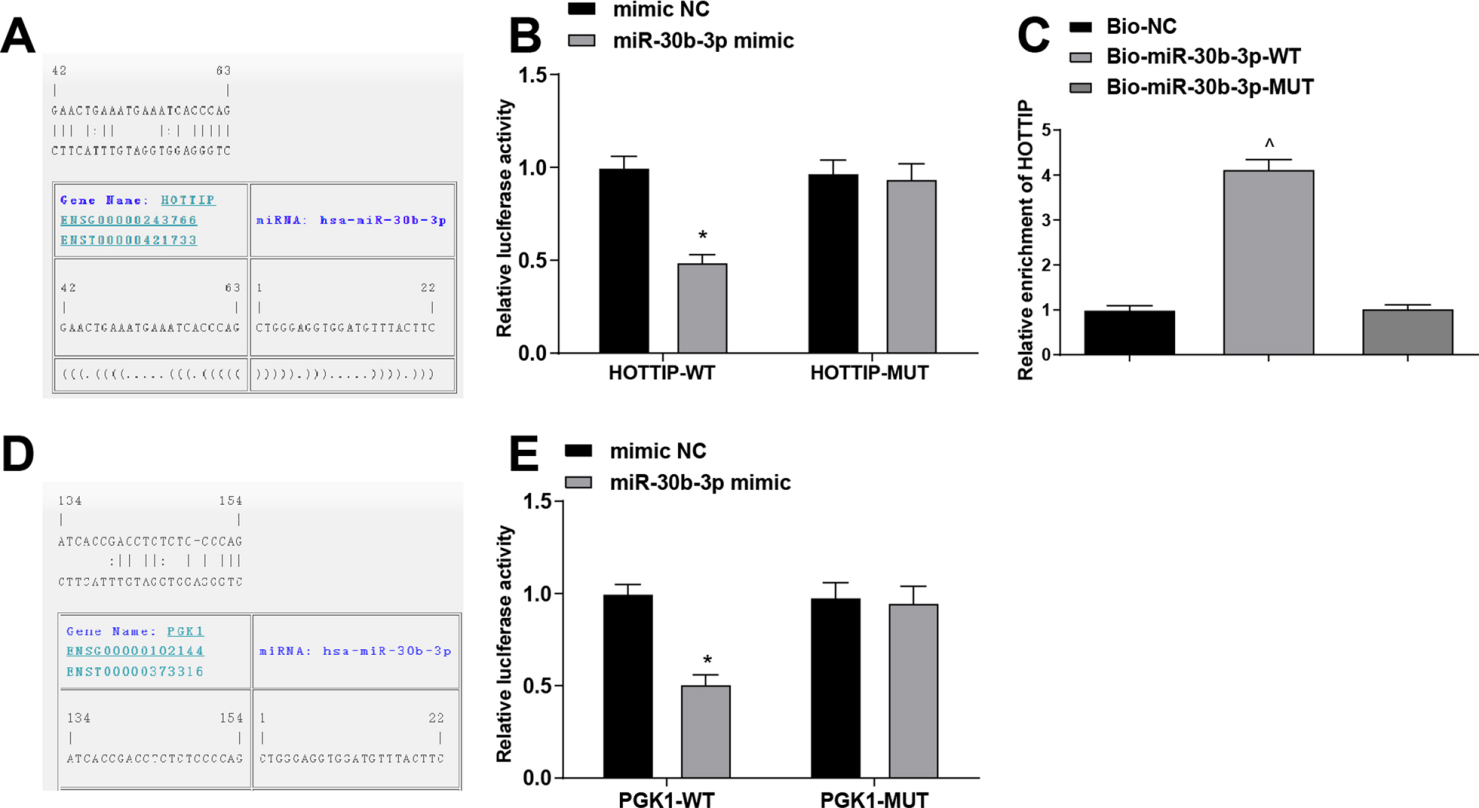
HOTTIP negatively regulates miR-30b-3p and PGK1 is targeted by miR-30b-3p. **A** Binding sites of HOTTIP and miR-30b-3p. **B** Binding relation between HOTTIP and miR-30b-3p was confirmed by dual-luciferase reporter gene assay. **C** Binding relation between HOTTIP and miR-30b-3p was confirmed by RNA pull-down assay. **D** Binding sites of miR-30b-3p and PGK1. **E** Target relation between miR-30b-3p and PGK1 was confirmed by dual-luciferase reporter gene assay. N = 3.*P < 0.01 *vs* the mimic NC group, ^*P* < 0.05 *vs* the Bio-NC group

To explore the regulatory relationship between HOTTIP and miR-30b-3p, a bioinformatics website RNA22 was used to predict their binding, and we found that there existed particular binding region between HOTTIP and miR-30b-3p (Fig. 4A), and the results of luciferase activity assay (Fig. 4B) indicated that the co-transfection of HOTTIP-WT and miR-30b-3p mimic reduced the luciferase activity of the ASFLSs, which was not evidently changed after co-transfected with HOTTIP-3’UTR MUT and miR-30b-3p mimic, indicating that there was a binding relation between HOTTIP and miR-30b-3p. The results from RNA pull-down assay (Fig. 4C) disclosed that HOTTIP was enriched by Bio-miR-30b-3p-WT, which exhibited no difference by Bio-miR-30b-3p-MUT, revealing that HOTTIP could bind with miR-30b-3p, thereby regulating its expression in ASFLSs.

In order to determine whether miR-30b-3p could target PGK1 to affect the cell processes, a bioinformatics software RNA22 was implemented to predict the targeting relation between miR-30b-3p and PGK1. It was predicted that miR-30b-3p could target PGK1 (Fig. 4D). As confirmed further (Fig. 4E), the co-transfection of PGK1-3’UTR WT and miR-30b-3p mimic reduced the luciferase activity, while it was not evidently altered after co-transfected with PGK1-3’UTR MUT and miR-30b-3p mimic. These data showed that miR-30b-3p could target PGK1.

### Silencing miR-30b-3p or overexpressing PGK1 reverses the improvement of AS by knocking down HOTTIP or up-regulating miR-30b-3p

**Fig. 5:**
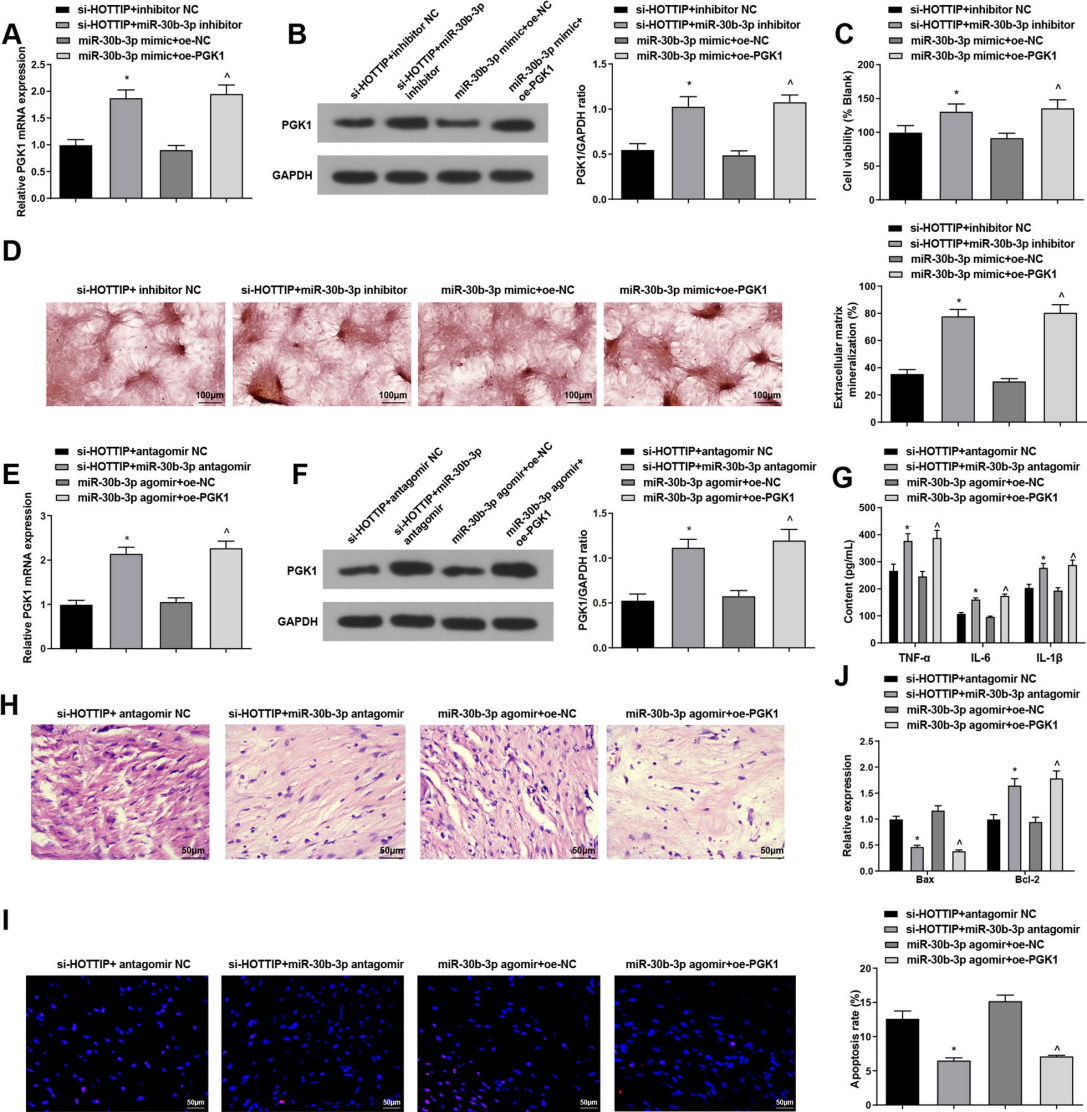
Silencing miR-30b-3p or overexpressing PGK1 reverses the improvement of AS by knocking down HOTTIP or up-regulating miR-30b-3p. **A**, **B** The validation of cell transfection. **C** Proliferation rate of ASFLSs was measured through MTT assay. D. The extracellular calcified nodules of ASFLSs were tested by Alizarin red staining. **E**, **F** Expression levels of HOTTIP and PGK1 in AS mice. **G** Contents of serum TNF-α, IL-6 and IL-β in each group. **H** Representative images of HE staining. **I** Representative images of TUNEL staining and the apoptosis rate of synoviocytes in each group. **J** Expression levels of Bax and Bcl-2 in synovial tissues. *P < 0.05 *vs* the si-HOTTIP + inhibitor NC group, ^*P* < 0.05 *vs* the miR-30b-3p mimic + oe-NC group

Finally, to explore whether HOTTIP could improve AS through the miR-30b-3p/PGK1 axis, we transfected si-HOTTIP + inhibitor NC, si-HOTTIP + miR-30b-3p inhibitor, miR-30b-3p mimic + oe-NC and miR-30b-3p mimic + oe-PGK1 into ASFLSs, and the transfection was successful (Fig. 5A, B). Subsequently, MTT, Alizarin red staining, ELISA, HE staining, TUNEL staining and other experiments were also performed (Fig. 5C–J), indicating that silencing miR-30b-3p or overexpressing PGK1 could reverse the improvement of HOTTIP knockdown or miR-30b-3p overexpression on AS through enhancing the proliferation ability and extracellular calcified nodules of ASFLSs, elevated the contents of TNF-α, IL-6 and IL-1β, aggravated the inflammatory infiltration and diminished the apoptosis rate of synoviocytes, thereby worsening the symptoms of AS.

In conclusion, HOTTIP can improve AS through the miR-30b-3p/PGK1 axis.

## Discussion

AS is a chronic inflammatory spondyloarthropathy which may develop to progressive spinal stiffness that ultimately makes patients susceptible to spinal fracture and spinal cord injury [3]. The ceRNA hypothesis indicated that lncRNAs could serve as ceRNAs to interact with miRNAs, thereby modulating the expression of mRNA [33]. Meanwhile, siRNAs could identify molecular targets and assess the curative effects of specific drugs and might be utilized for therapeutic purposes in tendon injuries and rheumatoid arthritis [34, 35]. The current work aimed to unearth the effects of the HOTTIP/miR-30b-3p/PGK1 axis in AS development, with the conclusion demonstrating that the overexpression of miR-30b-3p and inhibition of HOTTIP were able to suppress the proliferation and differentiation of ASFLSs via repressing PGK1, thus decelerating the progression of AS.

We firstly determined the expression levels of miR-30b-3p, HOTTIP and PGK1 in AS synovial tissues, normal synovial tissues, HFLSs and ASFLSs, and we have found that the levels of HOTTIP and PGK1 were up-regulated, while miR-30b-3p expression was decreased in AS synovial tissues and ASFLSs. These findings demonstrated the abnormal expression of HOTTIP, miR-30b-3p and PGK1 in AS. In line with the results, Li et al. have uncovered that HOTTIP is overexpressed in OS tissues and related to poor prognosis in OS patients [13], and it has been validated that HOTTIP expression is elevated in processes of endochondral ossification and osteoarthritic progression [14]. Furthermore, Sun et al. have clarified that miR-30c expression is decreased in OS lesions [36], and Zhao et al. have supported that PGK1 is highly expressed in synovial tissues of CIA rats [27]. Additionally, lncRNAs could serve as ceRNAs to interact with miRNAs, thereby modulating the expression of mRNA [33]. We have also found using bioinformatics prediction, dual-luciferase reporter gene assay, as well as RNA pull-down assay that HOTTIP could bind with miR-30b-3p, and PGK1 was targeted by miR-30b-3p. Similarly, it has been verified that HOTTIP could function as a molecular sponge to modulate miR-30b expression in ESCC [24]. However, the target relation of miR-30b-3p and PGK1 has not been investigated yet.

With the aim to identify the role of HOTTIP and miR-30b-3p in AS, the ASFLSs and AS mouse model were treated with altered HOTTIP and/or miR-30b-3p, and we found in our experiment that the knockdown of HOTTIP and restoration of miR-30b-3p impeded the proliferation of ASFLSs. In accordance with the finding, Zheng et al. have observed that the suppression of HOTTIP restrained the activation and proliferation of hepatic stellate cells (HSCs) in liver fibrosis [37], Li et al. have elucidated that the down-regulation of HOTTIP has the capacity to reduce the proliferation of trophoblast cells in preeclampsia [38], and it has also been clarified that the overexpressed miR-30 could suppress the proliferation of HSCs in liver fibrosis [39]. Another result of our research suggested that the degradation of HOTTIP and amplification of miR-30b-3p restrained the differentiation of ASFLSs. Consistently, Wa et al. have identified that miR-30b has the capacity to inhibit early chondrogenic differentiation of mouse embryo‑derived stem cells [40], and a former study has unveiled that the prostate cancer-derived PGK1 could induce osteoblastic differentiation of bone marrow stromal cells [41]. Additionally, we have figured out that the inhibited HOTTIP and increased miR-30b-3p enhanced the apoptosis of ASFLSs. Similar to this finding, Liu et al. have demonstrated that the decreased HOTTIP could accelerate the apoptosis of colorectal cancer cells [42], and a recent article has unraveled that the restoration of miR-30b-3p could diminish the apoptosis of lipopolysaccharide-treated alveolar epithelial cells in acute lung injury (ALI) [43]. The above data indicated the inhibitive roles of HOTTIP inhibition and miR-30b-3p amplification in ASFLS growth, revealing their suppressive effects on AS development.

Moreover, it has been unraveled that TNF-α, IL-6 and IL-1β are critical inflammatory indices in AS, which showed higher contents in AS patients versus healthy controls [44]. Thus, we determined the contents of TNF-α, IL-6 and IL-1β in tissues and cells. The results in our study indicated that the repression of HOTTIP and restoration of miR-30b-3p were able to suppress the inflammatory reaction in synovial tissues and synoviocytes in AS by inhibiting the levels of inflammatory factors. Consistent with this outcome, Mao et al. have unearthed that the knockdown of HOYYIP is able to restrict the development of osteoarthritis [45], and it has been discussed that the down-regulation of miR-30b-5p is accompanied with increase of TNF-α, IL-6 and IL-8 in ALI patients [46]. These findings contributed to identifying the role of HOTTIP and miR-30b-3p in inflammatory response in AS.

## Conclusion

The above data showed that the overexpressed miR-30b-3p or inhibited HOTTIP impedes the proliferation and differentiation of ASFLSs by suppressing PGK1, thereby restraining AS development. Therefore, HOTTIP could be used as a biomarker for AS, which may be helpful for exploring novel therapeutic strategies of AS. Our results elucidate the underlying mechanisms of AS and provide a marker for AS treatment in future. Of course, there is still much room for improvement in our research, such as the limited number of samples. In future studies, we will select a larger sample size to confirm our conclusions. Nevertheless, in-depth studies are warranted to investigate the detailed molecular mechanisms of HOTTIP, miR-30b-3p and PGK1 in AS.

## Supplementary Information

**Additional file 1**: **Table S1** Clinical characteristics and parameters of patients in the AS and the control groups. **Table S2** Primer sequences for PCR.

## Data Availability

The data that support the findings of this study are available from the corresponding author upon reasonable request.
