# Supplementary material for: LncRNA HOTTIP impacts the proliferation and differentiation of fibroblast-like synoviocytes in ankylosing spondylitis through the microRNA-30b-3p/PGK1 axis
Source: J Orthop Surg Res. 2023 Mar 24;18:237. doi: 10.1186/s13018-023-03653-4 (PMC10039568; doi:10.1186/s13018-023-03653-4)
Supplement: Supplementary file 1 — Additional file 1: Table S1 Clinical characteristics and parameters of patients in the AS and the control groups. Table S2 Primer sequences for PCR. [file 13018_2023_3653_MOESM1_ESM.docx]

**Supplementary Table 1** Clinical characteristics and parameters of patients in the AS and the control groups

| Parameter | Control | AS |
| --- | --- | --- |
| Age (years) | 58.6 ± 12.3 | 56.8 ± 10.61 |
| Gender (Male/Female) | 50/32 | 54/28 |
| BASFI |  | 2.1 ± 0.9 |
| BASDAI |  | 3.9 ± 1.4 |
| BASMI |  | 2.8 ± 1.0 |
| ASDAS-CRP |  | 2.85 ± 1.1 |
| Course of disease (years) |  | 14.3 ± 7.9 |
| HLA-B27 (n/%) |  | 74/90.00 |
| CRP (nmol/L) | 101.7 ± 54.2 | 14.0 ± 8.3 |
| ERS (mm/h) | 6.1 ± 3.5 | 16.9 ± 9.2 |

**Supplementary Table 2** Primer sequences for PCR

| Gene | Sequence (5’-3’) |
| --- | --- |
| miR-30b-3p | Forward: 5'- CTGGGAGGTGGATGTTTACTTC-3' |
|  | Reverse: universal primer |
| U6 | Forward: 5'-CTCGCTTCGGCAGCACATATACT-3' |
|  | Reverse: 5'-ACGCTTCACGAATTTGCGTGTC-3' |
| HOTTIP | Forward: 5'- AAGGGTCTCAGCTCCACAGA-3' |
|  | Reverse: 5'- CTGCCGTCTTTTCTGAGTCC-3' |
| PGK1 | Forward: 5'-GTGAAGGGGAAGCGGGTCGTCATGAGAG-3' |
|  | Reverse: 5'-GCTTGGAACAGCAGCCTTGATCCTCTGG -3' |
| Bax | Forward: 5‘-TTTTGCTACAGGGTTTCATCCAGG-3 ’ |
|  | Reverse: 5‘-ATCATCCTCTGCAGCTCCATATTG-3’ |
| Bcl-2 | Forward: 5‘-GGATAACGGAGGCTGGGATGC-3’ |
|  | Reverse: 5‘-ATTTGTTTGGGGCAGGTTTGTCG-3’ |
| GAPDH | Forward:5'-CCAGGGCTGCCATTTGCAGTGGCAAAGTGG-3' |
|  | Reverse: 5'-CCTGGAAGATGGTGATGGGCTTCCCGTTGA-3' |
